# Genome sequences of antimicrobial-resistant *Campylobacter coli* and *Campylobacter jejuni*, isolated from poultry in Ukraine

**DOI:** 10.1128/mra.00795-24

**Published:** 2024-11-21

**Authors:** Ihor Halka, Natalia Shchur, Eric Bortz, Svitlana Mandyhra, Vitalii Nedosekov, Orest Katsaraba, Ian Goodfellow, Devin M. Drown, Ganna Kovalenko

**Affiliations:** 1 Research Bacteriological Department, State Scientific Research Institute for Laboratory Diagnostics and Veterinary and Sanitary Expertise, Kyiv, Ukraine; 2 Faculty of Veterinary Medicine, National University of Life and Environmental Sciences of Ukraine, Kyiv, Ukraine; 3 Department of Biological Sciences, University of Alaska Anchorage, Anchorage, Alaska, USA; 4 Research Molecular and Genetical Department, State Scientific Research Institute for Laboratory Diagnostics and Veterinary and Sanitary Expertise, Kyiv, Ukraine; 5 Department of Obstetrics, Gynecology and Biotechnology of Animal Reproduction, Stepan Gzhytskyi National University of Veterinary Medicine and Biotechnologies Lviv, Lviv, Ukraine; 6 Department of Pathology, University of Cambridge, Cambridge, United Kingdom; 7 Department of Biology and Wildlife, University of Alaska Fairbanks, Fairbanks, Alaska, USA; 8 Institute of Arctic Biology, University of Alaska Fairbanks, Fairbanks, Alaska, USA; Wellesley College Department of Biological Sciences, Wellesley, Massachusetts, USA

**Keywords:** *Campylobacter jejuni*, *Campylobacter coli*, nanopore sequencing, genome analysis, AMR, Ukraine

## Abstract

Genomes of a *Campylobacter coli* OR12-like strain ChP2023 (1,713,995 bp) isolated from broiler chicken and *Campylobacter jejuni* subsp. *jejuni* NCTC 11168R-like strain KF2023 (1,729,995 bp) isolated from turkey, from poultry production facilities in Ukraine in 2023, were sequenced using Oxford Nanopore Technologies. Both genomes included antibiotic resistance genes and other virulence factors.

## ANNOUNCEMENT

We sequenced two *Campylobacter* spp. genomes isolated from the cecal contents randomly collected during the slaughter of poultry on farms to understand genetic diversity and antimicrobial resistance (AMR) in bacterial zoonoses in Ukraine (1–3). A *Campylobacter coli* strain ChP2023 was isolated from a broiler chicken in May 2023 in Cherkasy Oblast (49°04′14″N, 31°44′35″E) and a *Campylobacter jejuni* strain KF2023 was from a turkey in June 2023 in Kyiv Oblast (49°23′29″N, 30°11′30″E). Selective isolation followed ISO 10272-1:2017 (Procedure C) with direct plating on mCCD agar and incubation at 41.5°C for 24 h under microaerobic conditions (4, 5). Presumptive colonies were confirmed using the VITEK MS system (BioMerieux, France). The isolates were cryopreserved at –70°C. Bacterial biomass enrichment followed Procedure A: cultures were inoculated in Bolton broth with 5% lysed horse blood, incubated under microaerobic conditions at 37°C for 4 h and 41.5°C for 24 h (5). Cultures were then plated onto mCCD agar and incubated for another 24 h at 41.5°C before harvesting the colonies for DNA extraction.

DNA was extracted using IndiSpin Pathogen Kit (Indical Bioscience, Germany), yielding 16.8 ng/µL for *C. coli* and 2.73 ng/µL for *C. jejuni*. A library was prepared with the Rapid PCR Barcoding Kit (SQK-RPB004, Oxford Nanopore Technologies [ONT]) using barcodes BC01 and BC02 for the *C. coli* and BC03–BC05 for *C. jejuni*. Barcodes were pooled for each corresponding isolate and cleaned with AMPure XP beads (0.6×), resulting in final concentrations of 40.5 ng/µL for *C. coli* and 169.1 ng/µL for *C. jejuni*. A total of 1 µg of DNA (8 µL of *C. coli* and 4 µL of *C. jejuni*) was sequenced for 24 h on a GridION using an R9.4.1 flow cell.

The data were basecalled using Dorado version 7.2.13 (ONT) with the super-accuracy model (dna_r9.4.1_450bps_sup.cfg), filtering reads with a quality score of 10. Reads from barcodes corresponding to each species were then combined for downstream analysis (Table 1). Reads were assembled utilizing the EPI2ME wf-bacterial-genomes version 1.1.1 workflow (https://github.com/epi2me-labs/wf-bacterial-genomes). Briefly, this workflow used fastcat version 0.15.1 to combine input, Flye version 2.9.3 (6) for *de novo* assembly, Medaka version 1.11.3 (https://github.com/nanoporetech/medaka) for polishing, and MLST version 2.23.0 (https://github.com/tseemann/mlst) (7) for multilocus sequence typing (MLST). Default parameters were used except where otherwise noted. Each genome was analyzed by comprehensive genome analysis at BV-BRC version 1.040 (8) and annotated with PGAP version 6.7 (9) as part of the submission pipeline.

**TABLE 1:** Genomic characteristics of *Campylobacter coli* and *C. jejuni* isolates from poultry in Ukraine

Sequencing run report		
Total output	6,948,216,666 bp	
Total read count	2,151,311	
Total read *N*_50_	3,910 bp	

Assembly report		
Metrics	*C. coli* OR12-like strain ChP2023 *(BC01* and *BC02* combined)	*C. jejuni* subsp*. jejuni* NCTC 11168R-like strain KF2023 *(BC03, BC04,* and *BC05* combined)
SRA experiment accession	SRX25150463	SRX25150464
Sequencing yield (Gb)	1.59	1.02
Total reads number	400,976	215,867
Median read length (bp)	3,943	4,593
Median *Q* score	14	14
Contig count	4	9
Contigs *N*_50_ (bp)	1,035,987	359,068
Mean contig coverage depth (×)	915	386.14
Circular contigs number	0	2
Assembled genome size (bp)	1,713,995	1,729,995
GC content (%)	31.50	30.56
CDS	1,720	1,770
tRNA	50	48
rRNA	17	12
Completeness (%)	99.6	95.2
Contamination (%)	2.7	5.2
MLST^*a*^	2,273	Not determined
Antibiotic resistance genes	7	7
Virulence factors^*b*^	63	102

AMR mechanism	Putative genes	
Beta-lactam antibiotic inactivation enzymes^*c*^	OXA-61 family	OXA-184 family
	(bla-resistant)	
Efflux pump conferring antibiotic resistance	CmeABC, CmeDEF, MacB, YkkCD	CmeABC, CmeDEF, MacB, YkkCD
Antibiotic target replacement or absence of antibiotic target	7a-HSDH-like, *gidB*	7a-HSDH-like, *gidB*
Protein altering cell wall charge conferring antibiotic resistance	PgsA	PgsA
DNA topoisomerase (gyrase) subunit A genotype: prediction of ciprofloxacin resistance	gyrA: T86, D90	gyrA: T86I, D90
	(susceptible)	(resistant)

^*a*^ MLST, multilocus sequence typing.

^*b*^ Identified by the Virulence Factor Database (10).

^*c*^ Identified by the ResFinder prediction (11).

Genome distance analysis, using Mash (12) on BV-BRC showed that the *C. coli* strain ChP2023 grouped with *Campylobacter coli* strain OR12 (CP013733.1), an aerotolerant strain from a chicken farm (13). The *C. jejuni* strain KF2023 is closely related to *Campylobacter jejuni* subsp. *jejuni* NCTC 11168 (SZUC00000000.1), isolated from human infection.

PATRIC predicted genomic markers of AMR and virulence factors (8). AMR genes include multidrug efflux pumps that can impact resistance to fluoroquinolones, macrolides, and aminoglycosides (14–16). The *C. jejuni* strain KF2023 encoded a T86I mutation in *gyrA*, which in conjunction with the multidrug efflux pump CmeABC confers resistance to ciprofloxacin. Ciprofloxacin resistance has been observed in other *Campylobacter* sp. isolates from poultry in Ukraine and human infections and poultry in Europe (4, 14, 17–19).

## Data Availability

The genome project is indexed at GenBank under BioProject accession number PRJNA1129571. Genome assemblies are deposited under accession numbers JBEWFL000000000.1 and JBEWFM000000000.1. Direct SRA accessions for each data set are Campylobacter jejuni SRX25150464 and Campylobacter coli SRX25150463.
